# Distribution of *lag-1* Alleles, *ORF7*, and *ORF8* Genes of Lipopolysaccharide and Sequence-Based Types Among *Legionella pneumophila* Serogroup 1 Isolates in Japan and China

**DOI:** 10.3389/fcimb.2019.00274

**Published:** 2019-08-05

**Authors:** Luxi Jiang, Junko Amemura-Maekawa, Hongyu Ren, Yinan Li, Miho Sakata, Haijian Zhou, Miyo Murai, Bin Chang, Makoto Ohnishi, Tian Qin

**Affiliations:** ^1^State Key Laboratory for Infectious Disease Prevention and Control, Chinese Centre for Disease Control and Prevention, National Institute for Communicable Disease Control and Prevention, Beijing, China; ^2^Department of Respiratory Medicine, Zhejiang Provincial People's Hospital, People's Hospital of Hangzhou Medical College, Hangzhou, China; ^3^Department of Bacteriology I, National Institute of Infectious Diseases, Tokyo, Japan; ^4^Department of Health Sciences, Saitama Prefectural University, Saitama, Japan; ^5^Collaborative Innovation Center for Diagnosis and Treatment of Infectious Diseases, Hangzhou, China

**Keywords:** *Legionella pneumophila*, serogroup 1, lag-1 alleles, *ORF 7* and *ORF 8* genes, sequence-based typing

## Abstract

Approximately 85% of cases of Legionnaires' disease are caused by *Legionella pneumophila* serogroup 1. In this study, we analyzed the distribution of *lag-1* alleles, *ORF 7* and *ORF 8* genes of lipopolysaccharide (LPS) and sequence-based types of 616 *L. pneumophila* serogroup 1 strains isolated in Japan (206 clinical, 225 environmental) and China (13 clinical and 172 environmental). The *lag-1* gene was harbored by significantly more of the clinical isolates compared with the environmental isolates (90.3 vs. 19.1% and 61.6 vs. 3.0%, respectively; both *P* < 0.001). *ORF 7* genes were detected in 51.0% of Japanese clinical and 36.0% of Japanese environmental (*P* = 0.001) isolates, as well as 15.3% of Chinese clinical and 9.9% of Chinese environmental isolates (*P* = 0.544). *ORF 8* genes were detected in 12.1% of Japanese clinical and 5.8% of Japanese environmental (*P* = 0.017) isolates, as well as 7.7% of Chinese clinical and 3.4% of Chinese environmental isolates (*P* = 0.388). The Japanese and Chinese isolates were assigned to 203 and 36 different sequence-types (ST), respectively. ST1 was predominant. Most isolates with the same ST also had the same *lag-1, ORF 7*, and *ORF 8* gene subgroups. In conclusion, the *lag-1* was present in most of the clinical isolates, but was absent from most of the environmental isolates from both China and Japan, regardless of the water source and SBT type. PCR-based serotyping and subgrouping methods can be used to define a hierarchy of virulence genotypes that require stringent surveillance to prevent human disease.

## Introduction

*Legionella*, the causative agent of Legionnaires' disease, is a facultative intracellular Gram-negative bacteria that is ubiquitous in natural and man-made water systems (Rowbotham, 1980). These bacteria live parasitically in protozoa and can be found within biofilms (Rowbotham, 1980; Fields et al., 2002; Declerck, 2010; Stewart et al., 2012). *Legionella* is transmitted through inhaled aerosols and subsequently, enters and grows within human monocytes and alveolar macrophages, which can lead to a fatal form of pneumonia known as Legionnaires' disease (Isberg et al., 2009). To date, more than 60 species of *Legionella* and 70 serogroups (sg) have been identified (Viasus et al., 2019). Among them, *Legionella pneumophila* is the most common, causing ~90% of all cases of Legionnaires' disease (Viasus et al., 2019). At least 16 serogroups of *L. pneumophila* have now been identified, with serogroup 1 (sg1) predominating and causing approximately 85% of all cases of Legionnaires' disease (Fields et al., 2002; Yu et al., 2002; Carratala and Garcia-Vidal, 2010).

Lipopolysaccharide (LPS) is one of the most important outer membrane components and the major immune-dominant antigen for all the *Legionella* species (Ciesielski et al., 1986; Petzold et al., 2013). LPS consists of three parts: lipid A, core oligosaccharides, and polysaccharide (o) side-chains (Ciesielski et al., 1986). Furthermore, a genetic locus composed of at least 28 open reading frames (ORF) is essential in the biosynthesis of LPS core oligosaccharide and LPS polysaccharide O-chains (Petzold et al., 2013). The high diversity of LPS molecules provides the basis for classifying *L. pneumophila* into different serogroups and subgroups by detection using specific monoclonal antibodies (mAb) (Joly et al., 1983; Helbig et al., 1997, 2007). The Dresden Panel of mAbs, including mAb 3/1, was developed in 1997 to distinguish among heterogeneous bacterial groups belonging to *L. pneumophila* sg1 (Helbig et al., 2002). Moreover, the monoclonal types of sg1 were subdivided according to the presence or absence of the virulence-related epitope recognized by mAb 3/1. This epitope is not expressed by strains belonging to any of the other serogroups (Helbig et al., 2002). Surveys indicate that 65–100% of *L. pneumophila* sg1 clinical isolates react with mAb 3/1. In contrast, only 14.9–35% of *L. pneumophila* sg1 environmental isolates reacted with mAb 3/1 (Dournon et al., 1988; Harrison et al., 2007, 2009; Watkins et al., 2009). Mab 3/1 recognizes an epitope within the 8-*O-*acetylation group of the legionaminic acid of *L. pneumophila* sg1 LPS (Helbig et al., 1995; Zähringer et al., 1995; Zou et al., 1999). The *lag-1* gene encodes an *O*-acetyltransferase responsible for the 8-*O*-acetylation of legionaminic acid (Zou et al., 1999). Studies have shown that all mAb 3/1-postitive isolates harbor the *lag-1* gene, whereas mAb 3/1-negative isolates are either devoid of *lag-1* or contain missense mutations or insertions within this gene (Bernander et al., 2003).

The distribution of *lag-1* alleles and LPS genes in *L. pneumophila* sg1 isolates can be used to discriminate serogroups and monoclonal subgroups of the most common *L. pneumophila* serogroup 1. A previous study has established simple and rapid genotyping methods for culture-independent discrimination between serogroups of *L. pneumophila* and monoclonal subgroups of serogroup 1 (Thurmer et al., 2009). They identified a serogroup 1-specific genomic region, and developed two independent and suitable PCR assays for detecting serogroup 1 strains of *L. pneumophila* (Thurmer et al., 2009). Based on their research, we used conventional PCR methods to identify *L. pneumophila* sg1 subgroups in clinical and environmental isolates from Japan and China. We further characterized these isolates using the sequence-based typing (SBT) method, which is the most widely used molecular tool for epidemiological typing of *L. pneumophila* (Gaia et al., 2005; Ratzow et al., 2007).

## Materials and Methods

### Ethics

Ethical approval for this study was obtained from the meeting of ethics committee of National Institute for Communicable Disease Control and Prevention, China CDC.

### *L. pneumophila* sg1 Isolates

Two hundred and six clinical isolates were collected from the National Institute of Infectious Diseases, Japan (NIID, Japan). All the isolates were gathered from different patients from 30 prefectures between 2000 and 2015. There was no known epidemiologic linkage among these isolates. One hundred and ninety-eight of the isolates were from sporadic cases, and eight were from separate outbreaks occurring in seven prefectures. Two hundred and twenty-five environmental isolates were also collected from the NIID, Japan. The sources of these isolates were: bath water (92 isolates), cooling tower water (49 isolates), soil (36 isolates), shower water (30 isolates), and fountain (18 isolates).

Thirteen clinical isolates were collected from the Chinese Center for Disease Control and Prevention (China CDC). All the isolates were gathered from different patients, and they were obtained from six provinces between 1988 and 2016. All the isolates were sporadic cases. One hundred and seventy-two environment isolates were also collected from the China CDC. They were obtained from seven provinces between 2005 and 2016. The sources of these isolates were: air conditioner water (40 isolates), hot spring water (25 isolates), cooling tower water (65 isolates), and pipe water (42 isolates).

All these isolates were cultured on buffered charcoal yeast extract (BCYE) agar plates (Oxoid, Germany) for 48 h at 37°C under a 5% CO_2_ atmosphere.

### DNA Extraction and PCR Amplification

The target DNA of all the isolates were extracted using a QIAamp DNA Mini Kit (Qiagen, Germany) according to the manufacturer's instructions. The sequences of all the serogroup 1-specific and subgroup-specific primers used are listed in Supplementary Table S1. The “*lag-1*” primers were used for amplification of the internal *lag-1* fragment in all the stains. The “*lag-1*” primer pairs were used as “consensus” primers for all the strains. According to a previous study, *lag-1* can be subtyped into “*lag-1* Philadelphia,” “*lag-1* Knoxville,” and “*lag-1* Allentown” (Thurmer et al., 2009). The “intergenic region *ORF 6–8*,” “intergenic region *ORF 7–9*,” primers were used for amplification of “subgroup Benidorm/Bellingham” and “subgroup Knoxville” fragments, respectively, in the previous study (Thurmer et al., 2009). And in this study we named “intergenic region *ORF 6–8*,” “intergenic region *ORF 7–9*,” primers as “*ORF 7*,” “*ORF 8*,” respectively (listed in Supplementary Table S1). And the loci of “*ORF 7*,” “*ORF 8*” were shown in a schematic representation (Supplementary Figure S1). All PCRs were performed in thermal cyclers (SensoQuest LabCycler, Senso, Germany or LifeECO Thermal Cycler, BIOER, China) using Quick Taq HS DyeMix (Toyobo, Japan) by the following cycling parameters: denaturation at 94°C for 2 min; followed by 35 cycles of 94°C for 30 s, 55°C for 30 s, and 68°C for 1 min; and a final extension at 68°C for 7 min. The PCR products were resolved by 2.0% agarose gel electrophoresis and stained with GoldView or ethidium bromide, visualized under UV light, and analyzed using a Gel Doc system (BioRad, CA, USA).

### Reactivity With *lag-1, ORF 7*, and *ORF 8*

For *lag-1*-positive strains, the *lag-1* gene fragments were amplified by using the “*lag-1*” PCR primers shown in Supplementary Table S1. The *lag-1* gene fragments cannot be detected in *lag-1* (–) strains. The *lag-1*-positive strains were subtyped as “*lag-1* Philadelphia,” “*lag-1* Knoxville,” and “*lag-1* Allentown” using the primers shown in Supplementary Table S1 and the positive strains were designated “*lag-1*^*P*^,” “*lag-1*^*K*^,” and “*lag-1*^*A*^,” respectively, whereas the negative strains were designated “*lag-1*^*O*^.” That is the “*lag-1* Philadelphia,” “*lag-1* Knoxville,” and “*lag-1* Allentown” gene fragments cannot be detected in “*lag-1*^*O*^” strains (Tables 1–4).

**Table 1:** Japanese clinical isolates (*n* = 206) were divided into 15 subgroups of sg1.

**lag-1**	**Variation of** ***ORF 7*** **and** ***ORF 8***			**Total (%)**
	**Normal**	***ORF 7***	***ORF 8***	
*lag-1* (–)	4.9	3.9	0.9	9.7
*lag-1* (+)	32.0	47.1	11.2	90.3
*lag-1^*A*^*	24.8	22.3	8.7	55.8
*lag-1^*K*^*	2.4	24.8	1.5	28.7
*lag-1^*P*^*	4.4	0	1.0	5.4
*lag-1^*O*^*	0.4	0	0	0.4
Total %	36.9	51.0	12.1	100.0

**Table 2:** Japanese environmental isolates (*n* = 225) were divided into 15 subgroups of sg1.

***lag-1***	**Variation of** ***ORF 7*** **and** ***ORF 8***			**Total (%)**
	**Normal**	***ORF 7***	***ORF 8***	
*lag-1* (–)	52.0	24.0	4.9	80.9
*lag-1* (+)	6.2	12.0	0.9	19.1
*lag-1^*A*^*	4.4	3.1	0.9	8.4
*lag-1^*K*^*	0.9	8.9	0	9.8
*lag-1^*P*^*	0.9	0	0	0.9
*lag-1^*O*^*	0	0	0	0
Total %	58.2	36.0	5.8	100.0

**Table 3:** Chinese clinical isolates (n = 13) were divided into 15 subgroups of sg1.

***lag-1***	**Variation of** ***ORF 7*** **and** ***ORF 8***			**Total (%)**
	**Normal**	***ORF 7***	***ORF 8***	
*lag-1* (–)	23.1	15.3	0	38.4
*lag-1* (+)	53.9	0	7.7	61.6
*lag-1^*A*^*	23.1	0	0	23.1
*lag-1^*K*^*	0	0	0	0
*lag-1^*P*^*	7.7	0	0	7.7
*lag-1^*O*^*	23.1	0	7.7	30.8
Total %	77.0	15.3	7.7	100

**Table 4:** Chinese environmental isolates (*n* = 172) were divided into 15 subgroups of sg1.

***lag-1***	**Variation of** ***ORF 7*** **and** ***ORF***			**Total (%)**
	**Normal**	***ORF 7***	***ORF 8***	
*lag-1* (–)	85.5	8.7	2.8	97.0
*lag-1* (+)	1.2	1.2	0.6	3.0
*lag-1^*A*^*	0	0	0	0
*lag-1^*K*^*	0	0	0	0
*lag-1^*P*^*	0	0	0	0
*lag-1^*O*^*	1.2	1.2	0.6	3.0
Total %	86.7	9.9	3.4	100

All the strains were also subtyped by the “*ORF 7*” and “*ORF 8*” primers shown in Supplementary Table S1. The *ORF 7*-positive strains were defined as “variation of *ORF 7*” and the *ORF 8*-positive strains were defined as “variation of *ORF 8*,” respectively. Strains that were not detected by either “*ORF 7*” or “*ORF 8*” primers were defined as “normal *ORF 7* and *ORF 8*” (Tables 1–4).

All these PCR products were verified by sequencing.

### SBT

Genotyping was performed via the SBT protocol defined by the European Working Group for *Legionella* Infections (EWGLI) (www.ewgli.org). Genomic DNA was extracted and then amplified using primers targeting seven specific gene loci (i.e., *flaA, pilE, asd, mip, mompS, proA*, and *neuA*). Amplicons were sequenced with specific primers, and the resulting consensus sequences trimmed and compared to previously assigned allele numbers available on the website (http://www.hpa-bioinformatics.org.uk/legionella/legionella_sbt/php/sbt_homepage.php). According to a pre-determined order (i.e., *flaA, pilE, asd, mip, mompS, proA*, and *neuA*), the combination of alleles was defined as seven-digit allelic profile (e.g., 1, 1, 1, 1, 1, 1, and 1) and a sequence Type (ST) represented by a number (e.g., ST1). Newly identified STs were submitted to the EWGLI SBT database (http://www.hpa-bioinformatics.org.uk/legionella/legionella_sbt/php/sbt_homepage.php). Minimum spanning trees were constructed based on SBT profiles using BioNumerics 7.1 software (Applied Maths, Kortrijk, Belgium). A part of SBT profiles of some strains have been reported (Amemura-Maekawa et al., 2010, 2012, 2018).

### Statistical Analysis

Data were analyzed with the Pearson χ^2^ test using SPSS version 17.0 software (IBM SPSS, United States). When any expected number in the 2 × 2 contingency table was <5 and ≥1, the *P-*value was calculated using a continuity correction test. *P* < 0.05 was considered to indicate statistical significance.

### ATCC 33152

The DNA of *legionella pneumophila* type strain (pneumophila Philadelphia-1 ATCC 33152) was also extracted and amplified using primers listed in Supplementary Table S1.

## Results

### Subgroups of sg1 Isolates

**Figure 1:**
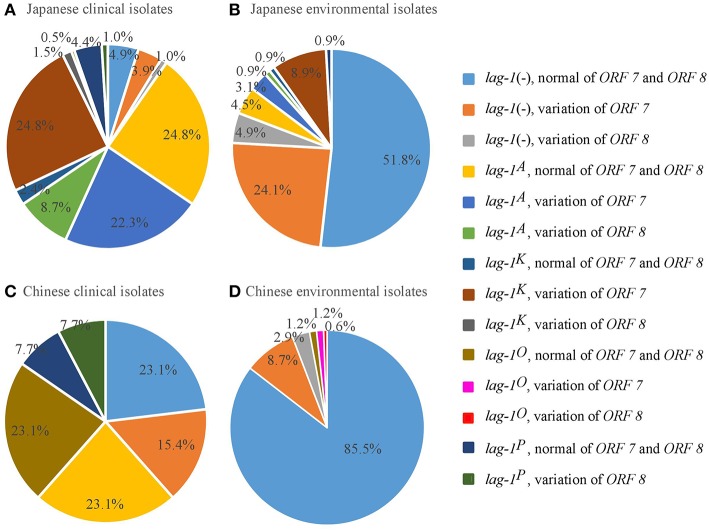
Distribution of *lag-1* alleles, and *ORF 7* and *ORF 8* genes of LPS among *L. pneumophila* serogroup 1 isolates from Japan and China. **(A)** Distribution of *lag-1* alleles, *ORF 7* and *ORF 8* genes of LPS among Japanese clinical isolates **(A)**, Japanese environmental isolates **(B)**, Chinese clinical isolates **(C)**, and Chinese environmental isolates **(D)**.

According to the reactivity with *lag-1, ORF 7*, and *ORF 8*, all the isolates were divided into 15 subgroups (Tables 1–4). Because the number of isolates for “lag-1, variation of ORF 8” is zero, there were 14 subgroups listed in Figure 1, finally.

Among the Japanese isolates, 90.3% of clinical isolates and 19.1% of the environmental isolates harbored the *lag-1* gene (*P* < 0.001 based on χ^2^ test on proportions) (Tables 1, 2, Figures 1A,B). Among the Chinese isolates, 61.6% of the clinical isolates and 3.0% of the environmental isolates harbored the *lag-1* gene (*P* < 0.001 based on χ^2^ test on proportions) (Tables 3, 4, Figures 1C,D). These results demonstrated that the *lag-1* was present in most of the clinical isolates but absent from most of the environmental isolates for both the Chinese and Japanese isolates.

Among the Japanese isolates, 51.0% of the clinical isolates and 36.0% of the environmental isolates harbored the *ORF 7* gene (*P* < 0.001 based on χ^2^ test on proportions) (Tables 1, 2, Figures 1A,B). Among the Chinese isolates, 15.3% of the clinical isolates and 9.9% of the environmental isolates harbored the *ORF 7* gene (*P* = 0.544 based on χ^2^ test on proportions) (Tables 3, 4, Figures 1C,D).

Among the Japanese isolates, 12.1% of clinical isolates and 5.8% of the environmental isolates harbored the *ORF 8* gene (*P* = 0.017 based on χ^2^ test on proportions) (Tables 1, 2, Figures 1A,B). Among the Chinese isolates, 7.7% of the clinical isolates and 3.4% of the environmental isolates harbored the *ORF 8* gene (*P* = 0.388 based on χ^2^ test on proportions) (Tables 3, 4, Figures 1C,D).

### Environmental Strains Classified According to Different Sources

According to the presence of the specific alleles of *lag-1, ORF 7, ORF 8*, and the different sources, all the environmental strains were classified and are listed in Supplementary Tables S2, S3. Only 2% (1/49) of Japanese cooling tower water isolates and 6% (4/65) of Chinese cooling tower water isolates harbored the *lag-1* gene. So as other environmental water sources. These data showed that, for both the Chinese and Japanese isolates, most of the environmental water isolates did not harbor the *lag-1* gene, regardless of the source.

### SBT Analysis

Based on SBT analysis, all the Japanese *L. pneumophila* sg1 isolates were assigned to 159 different STs (Supplementary Tables S4, S5) and all the Chinese *L. pneumophila* sg1 isolates were assigned to 36 different STs (Supplementary Tables S6, S7). Among these STs, ST1 was the most common for both the Japanese and Chinese isolates. For the clinical isolates, 2.4% (5/206) of the Japanese isolates and 0% of the Chinese isolates were classified as ST1. In contrast, for the environmental isolates, 35.6% (80/225) of the Japanese isolates and 57.6 % (99/172) of the Chinese isolates were classified as ST1.

**Figure 2:**
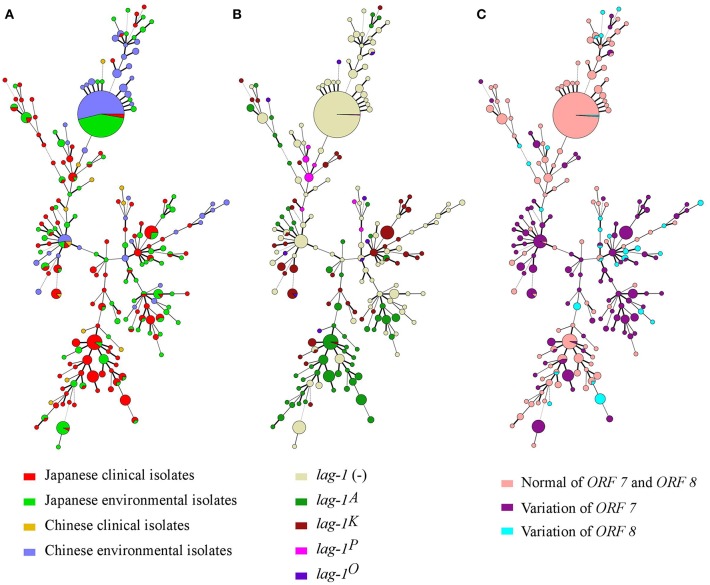
Minimum spanning tree analysis of 616 *L. pneumophila* serogroup 1 strains isolated in Japan and China. STs are shown as circles. The size of each circle indicates the number of isolates within this particular type. The 616 *L. pneumophila* serogroup 1 strains were divided into groups by countries and sources **(A)**, *lag-1* alleles **(B)**, and Distribution of *ORF 7* and *ORF 8* genes of LPS **(C)**.

**Figure 3:**
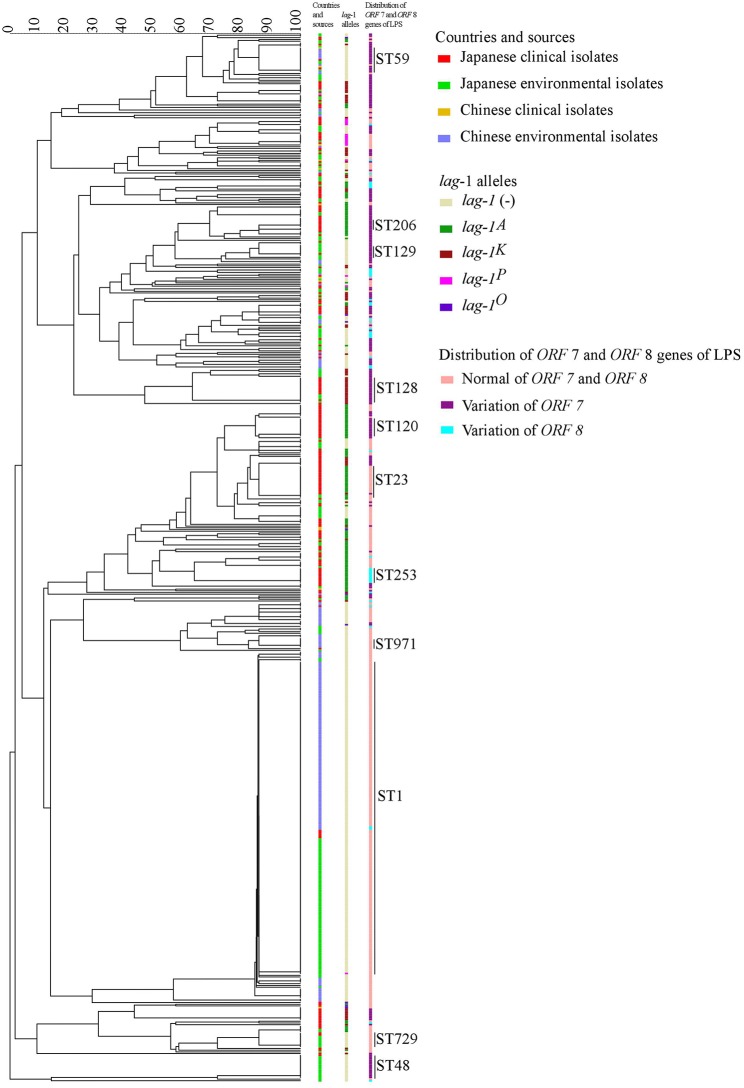
Evolutionary tree based on seven targeting specific gene loci. In the tree, the groups by countries and sources, lag-1 alleles and distribution of ORF 7 and ORF 8 genes of LPS were showed in different colors.

Minimum spanning trees were constructed based on the SBT profile grouped according to countries and sources (Figure 2A), *lag-1* subgroups (Figure 2B) and variations of *ORF 7* and *ORF 8* (Figure 2C). By comparing the three minimum spanning trees, we identified a correlation between ST and subgroups of sg1 (Figure 2). Most isolates with same ST also had the same *lag-1, ORF 7* and *ORF 8* gene subgroups. For example, all ST48 isolates had *ORF 7* variation but no *lag-1* gene; all ST42 isolates harbored the *lag-1*^*K*^ gene and *ORF 7* variation; all ST120 isolates harbored the *lag-1*^*A*^ gene and *ORF 7* variation; all ST23 isolates harbored the *lag-1*^*A*^ gene and normal *ORF 7* and *ORF 8* genes; most ST1 isolates were environmental isolates devoid of the *lag-1* gene and with normal *ORF 7* and *ORF 8* genes. This correlation between ST and subgroups was also presented by an evolutionary tree based on seven targeting specific gene loci (Figure 3).

### Subgroups of ATCC 33152

According to the reactivity with “*lag-1*,” “*lag-1*^*P*^,” “*lag-1*^*K*^,” “*lag-1*^*A*^,” “*ORF 7*,” and “*ORF 8*,” *legionella pneumophila* type strain ATCC 33152 harbored the *lag-1* gene, subtyped as “*lag-1*^*O*^,” and didn't harbor either *ORF 7 or ORF8* gene.

## Discussion

Previous studies suggest that LPS is the major immunodominant antigen for all the *Legionella* species (Ciesielski et al., 1986; Petzold et al., 2013). Furthermore, the *lag-1* gene encoding an *O*-acetyltransferase, which is responsible for the presence of the LPS epitope recognized by mAb 3/1, is exclusively found in sg1 strains (Petzold et al., 2013). Compared with other studies which used Dresden panel mentioned above, the other researchers usually used this method to differentiate the clinical sg1 strains into different subgroups (Helbig et al., 2002; Chasqueira et al., 2014). While, we used this method not only in clinical isolates but also in environmental strains, we also carried out comparative analysis. In our study, primers specific for the *lag-1* gene were tested for their ability to differentiate Chinese and Japanese isolates into *lag-1-*positive and *lag-1*-negative strains. In the present study, the *lag 1* gene was harbored by significantly more of the *L. pneumophila* sg1 clinical isolates than the environmental isolates, regardless of the country of origin. These findings are in accordance with a report of a study in the USA, in which 75% of the *L. pneumophila* sg1 clinical isolates but only 8% of environmental isolates harbored the *lag-1* gene (*P* = 0.0001 based on χ^2^ test on proportions) (Kozak et al., 2009). Thus, it can be speculated that the *lag-1* gene is present in the majority of clinical isolates, but is absent from most environmental isolates regardless of the country of origin, although the proportion of *lag-1*-positive clinical isolates varies in different countries. Furthermore, these results indicate that *L. pneumophila* sg1 infection exhibits unique virulence markers in every country. Due to the different national conditions in China and Japan, Legionellosis is not a legal infectious disease in China, and hospitals usually do not carry out clinical testing. Clinical strains are usually obtained through our active surveillance. Therefore, the number of Chinese clinical strains is lower.

In addition to *lag-1*, two intergenic regions expected to be specific for monoclonal subgroup Knoxville (*ORF 7*) and closely related subgroups Benidorm/Bellingham (*ORF 8*) were also used for selective genotyping. In a previous study in Germany (Thurmer et al., 2009), 31.7% (13/41) of the *L. pneumophila* sg1 clinical isolates and 50% (1/2) of the *L. pneumophila* sg1 environmental isolates harbored the *ORF 7* gene, with no significant difference between the two groups of isolates (*P* = 0.085 based on χ^2^ test on proportions). In accordance with this, there was no significant difference in the proportions of clinical and environmental Chinese *L. pneumophila* sg1 isolates harboring the *ORF 7* gene (15.3% of the clinical isolates and 9.9% of the environmental isolates; *P* = 0.544 based on χ^2^ test on proportions). In contrast, in the present study, a significantly higher proportion of the Japanese *L. pneumophila* sg1 clinical isolates harbored the *ORF 7* gene compared with the environmental isolates (51.4% of the clinical isolates and 36.0% of the environmental isolates; *P* = 0.001 based on χ^2^ test on proportions). These results show marked differences in the *ORF 7* characteristics of *L. pneumophila* sg1 isolates from these three countries, with a significant difference in the proportion of the clinical and environmental isolates harboring the *ORF 7* gene observed only in the Japanese isolates. In the German study mentioned previously, 26.8% (11/41) of the *L. pneumophila* sg1 clinical isolates and none (0/2) of the *L. pneumophila* sg1 environmental isolates harbored the *ORF 8* gene (*P* = 0.396 based on χ^2^ test on proportions). In our study, of the Japanese isolates, 12.1% of clinical isolates and 5.8% of environmental isolates harbored the *ORF 8* gene (*P* = 0.017 based on χ^2^ test on proportions). Of the Chinese isolates, 7.7% of clinical isolates and 3.4% of environmental isolates harbored the *ORF 8* gene (*P* = 0.388 based on χ^2^ test on proportions). Thus, similar to the observations for *ORF 7*, these results show marked differences in the *ORF 8* characteristics of *L. pneumophila* sg1 isolates from these three countries, with a significant difference in the proportion of the clinical and environmental isolates harboring the *ORF 8* gene observed only in the Japanese isolates. These results indicate that *L. pneumophila* sg1 infection exhibits unique virulence markers in Japan.

In addition, we carried out SBT analysis of the genetic relationship between *L. pneumophila* sg1 isolates from Japan and China. High SBT polymorphism was found in the analyzed strains. In accordance with previous reports on environmental *L. pneumophila* sg1 isolates (Amemura-Maekawa et al., 2012; Qin et al., 2014), ST1 was the dominant ST of environmental isolates in this study. Interestingly, we identified a correlation between STs and subgroups of sg1, as most isolates with same STs also had same *lag-1, ORF 7* and *ORF 8* gene subgroups. In addition, we observed that STs tend to associate with a single *lag-1* allele type. This suggests the existence of clonal groups of *L. pneumophila* sg1 that rarely recombine. Further studies are required to evaluate the possibility that particular combination of SBT and *lag-1* subgroups define a hierarchy of virulence genotypes that necessitates more stringent surveillance to prevent human diseases. Moreover, genome-wide sequencing techniques will facilitate the identification of *lag-1, ORF 7*, and *ORF 8* gene subgroups, so the highly virulent strains of *L. pneumophila* can be monitored and identified not only at the species and serogroup levels, but also at the genetic level.

## Data Availability

All datasets generated for this study are included in the manuscript/Supplementary Files.

## Author Contributions

LJ and JA-M carried out the experiments of this study, except those documented in Table 4 which were performed by HR and YL, and took part in writing the manuscript. MS and HZ provided materials and participated in data analysis and took part in writing the manuscript. MM, BC, and MO participated in data analysis and wrote the manuscript. TQ participated in conceiving and coordinating experimental work, and took part in writing the manuscript. And she also provided materials for this study. All authors read and approved the final manuscript.

### Conflict of Interest Statement

The authors declare that the research was conducted in the absence of any commercial or financial relationships that could be construed as a potential conflict of interest.
