# Supplementary material for: Distribution of lag-1 Alleles, ORF7, and ORF8 Genes of Lipopolysaccharide and Sequence-Based Types Among Legionella pneumophila Serogroup 1 Isolates in Japan and China
Source: Front Cell Infect Microbiol. 2019 Aug 5;9:274. doi: 10.3389/fcimb.2019.00274 (PMC6691400; doi:10.3389/fcimb.2019.00274)
Supplement: Supplementary file 1 [file Table_1.docx]

Supplementary table S1. Serogroup 1- and subgroup-specific primers

| Region^#^ | Forward and reverse primers and sequences (5’-3’) | Fragment size (bp) | reference |
| --- | --- | --- | --- |
| *lag-1* | lag-F: CTCACAACAAGTCAAGCAAC | 510 | [26] |
|  | lag-R: AAACCATACCAAAGCAACAT |  |  |
| *lag-1* Philadelphia | lag-1 Philadelphia-F: AGTGAAAGCGGATTTGGCA | 725 | [27] |
|  | lag-1 Philadelphia-R: TTAGCCACTCGCGAACTACG |  |  |
| *lag-1* Knoxville | lag-1 Knoxville-F: AGTATGGGTGGATTTGGTG | 725 | [27] |
|  | lag-1 Knoxville-R: TCATCCACTCACGAACCAGT |  |  |
| lag-1 Allentown | lag-1 Allentown-F: AGTGGAGGCAGAGTTGGCA | 725 | [27] |
|  | lag-1 Allentown-R: TTAGCCACTCGCGAACTACG |  |  |
| ORF7 | Lens 6-8 do: GATACTAACAGCCAAGGTG | 979 | [27] |
|  | Lens 7 up1: TCCAACCCAAGGAATTCCTG |  |  |
| ORF8 | Corby 7-9 up: CTAGATCACATTCGTATCGTC | 1010 | [27] |
|  | Corby 7-9 do: GTTGACGAGATTGTATCTC |  |  |

^#^Regions are as shown in Supplementary Figure S1.

Supplementary table S2. 225 Japanese environmental isolates were classified by different sources

| Sources | *lag-1* | Variation of *ORF 7* and *ORF 8* | | | Total No. |
| --- | --- | --- | --- | --- | --- |
|  |  | Normal | *ORF 7* | *ORF 8* |  |
| Cooling tower water (49) | *lag-1* (-) | 43 | 4 | 1 | 48 |
|  | *lag-1* (+) | 1 | 0 | 0 | 1 |
| Bath water(92) | *lag-1* (-) | 20 | 30 | 10 | 60 |
|  | *lag-1* (+) | 5 | 25 | 2 | 32 |
| Soil (36) | *lag-1* (-) | 19 | 12 | 0 | 31 |
|  | *lag-1* (+) | 4 | 1 | 0 | 5 |
| Shower water (30) | *lag-1* (-) | 21 | 5 | 0 | 26 |
|  | *lag-1* (+) | 3 | 1 | 0 | 4 |
| Fountain (18) | *lag-1* (-) | 14 | 3 | 0 | 17 |
|  | *lag-1* (+) | 1 | 0 | 0 | 1 |

Supplementary table S3. 172 Chinese environmental isolates were classified by different sources

| Sources | *lag-1* | Variation of *ORF 7* and *ORF 8* | | | Total No. |
| --- | --- | --- | --- | --- | --- |
|  |  | Normal | *ORF 7* | *ORF 8* |  |
| Cooling tower water (65) | *lag-1* (-) | 50 | 9 | 2 | 61 |
|  | *lag-1* (+) | 2 | 1 | 1 | 4 |
| Air conditioning water (40) | *lag-1* (-) | 33 | 5 | 2 | 40 |
|  | *lag-1* (+) | 0 | 0 | 0 | 0 |
| Hot spring water (25) | *lag-1* (-) | 22 | 1 | 1 | 24 |
|  | *lag-1* (+) | 0 | 1 | 0 | 1 |
| Pipe water (42) | *lag-1* (-) | 42 | 0 | 0 | 42 |
|  | *lag-1* (+) | 0 | 0 | 0 | 0 |

Supplementary table S4. 206 Japanese clinical isolates characterized by SBT

| *lag-1* | Variation of *ORF 7* and *ORF 8* | | |
| --- | --- | --- | --- |
|  | Normal | ORF 7* | ORF 8* |
| *lag-1* (-) | ST1 (5) ^#^, ST593 (1), ST608 (1), ST739 (2), ST954 (1) | ST48 (1), ST59 (2), ST129 (1), ST278 (1), ST763 (1), ST1857 (1), ST1964 (1) | ST595 (1), ST839 (1) |
| *lag-1^A^* | ST23 (16), ST118 (1), ST132 (3), ST140 (1), ST224 (1), ST294 (2), ST298 (1), ST299 (1), ST300 (1), ST352 (2), ST506 (1), ST507 (4), ST550 (3), ST594 (1), ST613 (1), ST679 (1), ST850 (1), ST876 (2) ST891 (1), ST905 (2), ST1187 (1), ST1846 (1), ST1847 (1), ST1865 (1), ST2128 (1) | ST89 (2), ST120 (11), ST122 (2), ST139 (2), ST142 (3), ST256 (1), ST301 (1), ST306 (6), ST309 (1), ST507 (3), ST566 (3), ST623 (1), ST644 (2), ST682 (1), ST701 (1), ST746 (1), ST843 (1), ST1798 (1), ST1867 (1), ST1924 (1), ST2126 (1) | ST2 (1), ST353 (9), ST624 (1), ST642 (3), ST876 (1), ST1187 (1), ST1933 (1), ST2127 (1) |
| *lag-1^K^* | ST141 (1), ST288 (1), ST307 (1), ST348 (1), ST1480(1) | ST2 (1), ST23, ST42 (7), ST84(1), ST138 (11), ST180(1), ST208(1), ST384 (5), ST502 (4), ST505 (4), ST530 (1), ST538 (1), ST552 (1), ST553 (1), ST612 (1), ST622 (1), ST686 (1), ST687 (2), ST688 (1), ST769 (1), ST882 (1), ST947 (1), ST1186 (1), ST1756 (1) | ST531, ST1845, ST1965(1) |
| *lag-1^P^* | ST211 (3), ST609 (5), ST1773 (1) |  | ST9(1), ST551(1) |
| *lag-1^0^* | ST143 (1) |  |  |

^#^Numbers in the brackets represent the number of isolates for different STs

Supplementary table S5. 225 Japanese environmental isolates characterized by SBT

| *lag-1* | Variation of *ORF 7* and *ORF 8* | | |
| --- | --- | --- | --- |
|  | Normal | *ORF 7* | *ORF 8* |
| *lag-1* (-) | ST1 (79) ^#^, ST10 (1), ST22 (7), ST45 (1), ST52 (2), ST260, ST445 (2), ST448 (2), ST561 (1), ST593 (2), ST607 (1), ST739 (7), ST954 (2), ST974 (1), ST1008 (1), ST1030 (1), ST1077 (1), ST1290 (1), ST1426 (1), ST1763 (1), ST2061 (2) | ST48 (13), ST59 (3), ST86 (2), ST127 (4), ST128 (2), ST129 (6), ST138 (1), ST201 (1), ST278 (1), ST493 (1), ST545 (1), ST552 (2), ST599 (2), ST600 (1), ST601 (1), ST603 (1), ST604 (1), ST605 (1), ST763 (1), ST977 (2), ST981 (1), ST1027 (1), ST1144 (1), ST1151 (1), ST1209 (1), ST1544 (1), ST1859 (1) | ST305 (2), ST604 (1), ST980 (2), ST982 (1), ST1717 (1), ST1835 (1), ST2093 (1), ST2099 (2) |
| *lag-1^A^* | ST23 (2), ST132 (1), ST352 (2), ST679 (2), ST741 (1), ST876 (1), ST976 (1) | ST89 (1), ST122 (1), ST131 (1), ST256 (1), ST566 (2), ST610 (1) | ST2 (1), ST642 (1) |
| *lag-1^K^* | ST343 (1), ST740 (1) | ST137 (1), ST138 (4), ST162 (1), ST165 (1), ST302 (1), ST448 (1), ST505 (1), ST530 (1), ST622 (1), ST687 (3), ST979 (1), ST1428 (1), ST1545 (1), ST2000 (2) |  |
| *lag-1^P^* | ST1 (1), ST609 (1) |  |  |

^#^Numbers in the brackets represent the number of isolates for different STs

Supplementary table S6. 13 Chinese clinical isolates characterized by SBT

| *lag-1* | Variation of *ORF 7* and *ORF 8* | | |
| --- | --- | --- | --- |
|  | Normal | *ORF 7* | *ORF 8* |
| *lag-1* (-) | ST1439 (1) ^#^, ST1440 (1), ST1999 (1) | ST59 (2) |  |
| *lag1^A^* | ST-2344 (1), ST-2366 (1), ST-2369 (1) | 0 | 0 |
| *lag1^K^* | 0 | 0 | 0 |
| *lag1^P^* | ST36 (1) | 0 | ST742 (1) |
| *lag-1^0^* | ST42 (1), ST2345(1), ST-2368 (1), |  |  |

^#^Numbers in the brackets represent the number of isolates for different STs

Supplementary table S7. 172 Chinese environmental isolates characterized by SBT

| *lag-1* | Variation of *ORF 7* and *ORF 8* | | |
| --- | --- | --- | --- |
|  | Normal | *ORF 7* | *ORF 8* |
| *lag-1* (-) | ST1 (99) ^#^, ST154 (1), ST269 (1), ST345 (2), ST354 (1), ST595 (2), ST630 (1), ST719 (3), ST752 (6), ST971 (6), ST986 (2), ST1119 (1), ST1177 (1), ST1471 (3), ST1556 (2), ST1559 (2), ST1560 (2), ST1561 (2), ST1562 (1), ST1563 (2), ST1565 (1), ST1566 (1), ST1656 (3), ST1657 (1), ST1672 (1), ST1675 (1) | ST59 (8), ST60 (1), ST199 (2), ST454 (2), ST1557 (1), ST1558 (1), ST1562 (1) | ST1 (2), ST1021 (2), ST1564 (1) |
| *lag-1^A^* | 0 | 0 | 0 |
| *lag-1^K^* | 0 | 0 | 0 |
| *lag-1^P^* | 0 | 0 | 0 |
| *lag-1^0^* | ST583 (1), ST1665 (1) | ST1562 (1), ST1728 (1) | ST345 (1) |

^#^Numbers in the brackets represent the number of isolates for different STs
