# Supplementary material for: Distribution of lag-1 Alleles, ORF7, and ORF8 Genes of Lipopolysaccharide and Sequence-Based Types Among Legionella pneumophila Serogroup 1 Isolates in Japan and China
Source: Front Cell Infect Microbiol. 2019 Aug 5;9:274. doi: 10.3389/fcimb.2019.00274 (PMC6691400; doi:10.3389/fcimb.2019.00274)
Supplement: Supplementary file 2 [file Image_1.pdf]

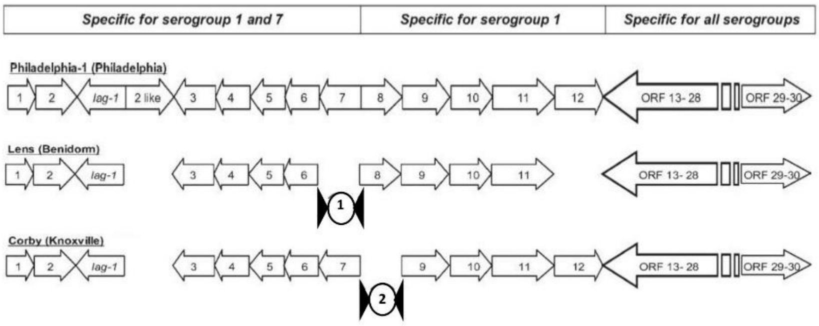

Supplementary Figure S1 Schematic representation of regions of the LPS synthesis cluster in published L. pneumophila serogroup 1 strains and localization of the serogroup1-specific (indicated by arrowheads) and subgroup-specific primer pairs (indicated by arrowheads and circled numbers 1, 2), see also Table S1. The regions are: ① Benidorm/Bellingham ORF 6–8, ② Knoxville ORF 7–9.
